# Cenobamate in Dravet syndrome with electrophysiologically confirmed *SCN1A* loss-of-function variants: long-term real-world follow-up

**DOI:** 10.3389/fphar.2026.1894717

**Published:** 2026-09-23

**Authors:** Pascal Fenske, Astghik Abrahamyan, Konstantin L. Makridis, Sabrina Pommer, Thorsten Trimbuch, Anna-Lena Friedo, Christoph Kellinghaus, Florian-Phillipp Losch, Bettina Schmitz, Christian Boßelmann, Shyamala Mani, Christian Rosenmund, Angela M. Kaindl

**Affiliations:** 1 Department of Pediatric Neurology, Charité – Universitätsmedizin Berlin, Berlin, Germany; 2 Center for Chronically Sick Children, Charité – Universitätsmedizin Berlin, Berlin, Germany; 3 Institute of Cell- and Neurobiology, Charité – Universitätsmedizin Berlin, Berlin, Germany; 4 German Epilepsy Center for Children and Adolescents, Charité – Universitätsmedizin Berlin, Berlin, Germany; 5 Section CNS Development and Neurologic Disease, German Center for Child and Adolescent Health (DZKJ), Berlin, Germany; 6 Institute of Neurophysiology, Charité – Universitätsmedizin Berlin, Berlin, Germany; 7 Epilepsieklinik Tabor, Bernau, Germany; 8 Epilepsy Center Münster-Osnabrück, Klinikum Osnabrück, Osnabrück, Germany; 9 Department of Neurology, Vivantes Humboldt-Klinikum, Berlin, Germany; 10 Department of Neurology and Epileptology, Hertie Institute for Clinical Brain Research, Eberhard Karls University of Tübingen, Tübingen, Germany

**Keywords:** anti-seizure medication, cenobamate, developmental and epileptic encephalopathy, Dravet syndrome, epilepsy

## Abstract

Dravet syndrome (DS) is a severe developmental and epileptic encephalopathy (DEE) characterized by seizures and developmental delay/regression, primarily caused by loss-of-function (LoF) variants in the *SCN1A* gene, which encodes the Nav1.1 sodium channel. Hyperexcitability in DS results from impaired inhibitory interneuron function. Sodium channel blockers are considered to be contraindicated particularly in the long-term treatment of patients with LoF variants in sodium channels as they may exacerbate seizures. However, we previously reported that cenobamate (CNB), an anti-seizure medication thought to be contraindicated due to its sodium channel blocking action, was effective in treating adult DS patients. These patients displayed a typical DS phenotype. Extended long-term follow-up demonstrated sustained clinical benefit of CNB treatment. To functionally characterize the previously unclassified patient-specific *SCN1A* variants, we conducted electrophysiological and localization studies using HEK cells expressing Nav1.1. Whole-cell patch-clamp recordings confirmed that the patient variants were LoF, ranging from markedly reduced to nearly absent peak sodium currents. Acute CNB exposure did not alter peak sodium current density under the experimental conditions used. These findings demonstrate sustained clinical benefit of CNB in adults with genetically confirmed DS carrying electrophysiologically confirmed LoF SCN1A variants and further expand the clinical experience with CNB in this patient population.

## Introduction

Dravet syndrome (DS) is a severe developmental and epileptic encephalopathy (DEE) characterized by drug-resistant seizures and developmental delay as well as regression. DS is primarily caused by variants in the *SCN1A* gene, which encodes the α subunit of the voltage-gated sodium channel Nav1.1 (Dravet, 2011; Chiron and Dulac, 2011). These variants typically result in a loss-of-function (LoF) of inhibitory interneurons, leading to hyperexcitability and thus seizures (Yu et al., 2006; Catterall, 2018; Berecki et al., 2019). Given this underlying mechanism, known as the interneuron hypothesis, sodium channel blockers are thought to be contraindicated in DS as they may exacerbate seizures and can aggravate developmental regression (Guerrini et al., 1998; Dalic et al., 2015).

Cenobamate (CNB) is a novel antiseizure medication (ASM) approved for the treatment of adults with focal drug-resistant epilepsy (DRE), demonstrating unexpectedly high rates of seizure freedom or significant seizure reduction, and high retention rates (Makridis et al., 2022a; Makridis and Kaindl, 2023). CNB is believed to have a dual mechanism of action: (i) acting as a positive allosteric modulator of GABA_A_ receptors and (ii) blocking persistent sodium currents (Nakamura et al., 2019). Given its sodium channel mechanism, CNB would typically be contraindicated particularly for the long-term treatment of patients with DS. However, we previously reported four adult DS patients who experienced significant seizure reduction with CNB treatment, with an observation period of up to 542 days (median 428.5 days; Makridis et al., 2022b).

Building on our previously reported clinical observations, we extended the long-term follow-up of four adults with genetically confirmed Dravet syndrome treated with cenobamate to further evaluate treatment durability and long-term safety. Because the patient-specific SCN1A variants had not previously been functionally characterized and were only predicted by *in silico* analyses to result in loss of function, we performed electrophysiological and localization studies to determine their functional consequences and provide biological context for the clinical observations.

## Methods

In this retrospective, multi-center follow-up study, previously reported patients were systematically re-evaluated. Seizure frequency and treatment data were extracted retrospectively from treating physicians’ documentation in the electronic and paper-based medical records, which were based on patient and caregiver reports (approval numbers: EA2/084/18 and 198/2010BO1).

### Cell culture

HEK 293T cells were cultured in DMEM with 10% FBS, penicillin, and streptomycin, maintained at 37 °C with 5% CO_2_ and 100% humidity. Transfection was performed using Lipofectamine 3000 (Life Technologies GmbH, Darmstadt, Germany). The wild-type SCN1A coding sequence was derived from the human Nav1.1 transcript NM_001165963 (GenBank) and obtained through the pIR-CMV-IRES-mScarlet plasmid backbone (Addgene #162278). SCN1A patient variants were generated by site-directed mutagenesis, PCR amplification, and Gibson assembly. Construct integrity was verified by restriction digestion and sequencing. The resulting expression plasmids included pIR-CMV-SCN1A-D1288Y-IRES-mScarlet (Pat1), pIR-CMV-SCN1A-Y202D-IRES-mScarlet (Pat2), pIR-CMV-SCN1A-L780Stop-IRES-mScarlet (Pat3), and pIR-CMV-SCN1A-C1588R-IRES-mScarlet (Pat4).

### Localization and protein expression studies

HEK293T cells expressing WT or mutant SCN1A channels were analyzed by immunofluorescence microscopy to assess subcellular localization. Image acquisition and colocalization analysis were performed blinded to the experimental condition using samples from three independent cell cultures. Colocalization with the plasma membrane marker SPATPase and the endoplasmic reticulum marker calnexin was quantified using the JACoP plugin in ImageJ. Pearson’s correlation coefficients were calculated at the single-cell level. For each coverslip, cells from 2 to 3 fields of view were analyzed and averaged to obtain one coverslip-level value. Individual coverslips are shown as data points. Primary antibodies used were *SCN1A* (NeuroMab 75-023), Calnexin (Millipore #C4731-.2ML), and Sodium Potassium ATPase (Invitrogen MA5-32184), followed by AlexaFluor 680 and AlexaFluor 488 conjugated secondary antibodies.

For qualitative assessment of SCN1A protein expression by Western blot, HEK cells were lysed 48 h post-transfection using the Mem-PER™ Plus Membrane Protein Extraction Kit (Thermo Fisher Scientific). Membrane fractions were isolated, subjected to SDS-PAGE, and transferred to nitrocellulose membranes. After blocking with 5% skim milk in PBS-T, membranes were incubated overnight at 4 °C with primary antibodies against *SCN1A* and Sodium Potassium ATPase. Detection was performed using horseradish peroxidase-conjugated secondary antibodies, followed by ECL using the Bio-Rad system and visualized with an Azure 600 system. Western blot analysis was performed as a qualitative assessment of SCN1A protein expression. Because only a single representative experiment was available, no quantitative densitometric analysis was performed.

### Electrophysiological experiments

HEK cells were transfected with WT or mutant *SCN1A* constructs (D1288Y, Y202D, L780*, C1588R). Whole-cell patch-clamp recordings were conducted to assess sodium current properties using a Multiclamp 700B amplifier (Molecular Devices). Currents were recorded 24–48 h post-transfection. Series resistance was compensated by 70%, and only cells with resistances <12 MΩ were analyzed. Data were acquired at 10 kHz using pClamp 10 software and filtered with a 3 kHz low-pass Bessel filter. Offline analysis was done using AxographX and pClamp software. Transfected cells were identified by red fluorescence, and recordings were done at room temperature. The bath solution contained 145 mM NaCl, 5 mM KCl, 1.8 mM CaCl2, 1 mM MgCl2, and 10 mM Hepes, pH 7.4. The internal solution was 40 mM CsCl, 10 mM NaCl, 80 mM CsF, 11 mM EGTA, 10 mM Hepes, and 1 mM CaCl2, pH 7.3. Voltage-dependent parameters were measured with a holding potential of −90 mV. The standard IV protocol involved 500 ms pulses from −90 mV to +60 mV.

Acute CNB treatment was performed by rapid solution exchange using a flow-pipe system positioned above the recorded cell, enabling millisecond exchange of extracellular solution and immediate exposure to 300 μM CNB.

### Statistical analysis

Statistical analysis was performed using GraphPad Prism 9. Data were obtained from at least three independent cultures. Group comparisons were conducted using Kruskal–Wallis tests followed by Dunn’s *post hoc* correction. Normality was assessed using the Shapiro–Wilk test and variance homogeneity using Levene’s test, both confirming the appropriateness of a non-parametric approach. Statistical significance was defined as p < 0.05.

## Results

We extended the clinical follow-up of the four previously reported adults with genetically confirmed Dravet syndrome treated with CNB to evaluate the long-term durability of treatment response and safety. All four patients remained on CNB throughout follow-up, resulting in a retention rate of 100%.

**Patient 1 (p.Asp1288Tyr)** had a pre-CNB seizure frequency of 8 GTCS per month treated with levetiracetam and clobazam (CLB). Due to excessive daytime sedation, CLB was discontinued, and the patient continued on CNB monotherapy (125 mg–0–250 mg). This adjustment alleviated the sedation issues. Also, the patient’s previous behavioral problems resolved, allowing for a transition to assisted living accommodation with his newfound functional independence. While daytime seizure freedom was successfully achieved, the patient continued to experience nocturnal generalized tonic-clonic seizure (GTCS) 3-4 times per month (>80% seizure reduction) (follow-up time 43 months). Due to the increased risk of sudden unexpected death in epilepsy (SUDEP) associated with nocturnal seizures and GTCS, the patient was admitted for inpatient evaluation in August 2024. During this admission, valproic acid (VPA) was introduced and titrated to a target dose of 1,000 mg**. Patient 2 (p.Tyr202Asp)** had experienced five GTCS per week prior to treatment with CNB. At the time of reevaluation 44 months into CNB treatment (200 mg/d), she experienced only one seizure every 6 months. CNB was given as an add-on in addition to topiramate and phenobarbital. Zonisamide had been successfully tapered off after initiation of CNB, resolving the initial problem of fatigue. Patient 3 (p.Leu780*) had a favorable response to treatment with 200 mg/d CNB in addition to VPA and brivaracetam. His pre-cenobamate baseline seizure frequency of 5–10 per month was reduced to 2 seizures per month under treatment with CNB at follow-up 26 months into treatment with CNB. In the course of a suspected aspiration pneumonia, he experienced a decline in seizure control with 3–4 GTCS per month and multiple daily focal seizures. Empirical antibiotic therapy with ampicillin/sulbactam, later escalated to piperacillin/tazobactam, ameliorated the infection and correspondingly reduced seizure frequency to baseline 1 month after discharge. The patient was reported to have succumbed to SUDEP at home. This outcome reflects the known SUDEP risk associated with DS despite treatment response. **Patient 4 (p.Cys1588Arg)** who had exhibited a pre-cenobamate baseline seizure frequency of 20 per month initially achieved seizure freedom for 34 months on 200 mg/d CNB. He then experienced approximately three seizures per week. The treating physicians refrained from further dose escalation given the significant daytime fatigue and drowsiness reported by the patient since starting CNB.

To provide an integrated overview of the cohort, the clinical characteristics and long-term treatment outcomes of all four patients are summarized in Table 1.

**TABLE 1:** Clinical characteristics, SCN1A variants, and long-term cenobamate (CNB) treatment outcomes of the four patients. Detailed baseline characteristics of this cohort have been reported previously (Makridis et al., 2022b). Abbreviations: SUDEP, Sudden Unexpected Death in Epilepsy.

Parameter	Patient 1	Patient 2	Patient 3	Patient 4
SCN1A mutation	p.Asp1288Tyr	p.Tyr202Asp	p.Leu780*	p.Cys1588Arg
Sex	Male	Female	Male	Male
Alive/deceased	Alive	Alive	Deceased (suspected SUDEP)	Alive
Age at seizure onset (months)	3	5	8	8
Age at CNB initiation (years)	21.8	26.8	26.0	22.0
Final CNB dose (mg/day)	375 mg	200 mg	200 mg	200 mg
Total follow-up time (months)	43	44	26 (died thereafter)	34
Seizure reduction (%)	>80%	>80%	>80% until death	>90%
Adverse effects	Fatigue	Fatigue	None	Fatigue and drowsiness
Electrophysiological classification	Loss-of-function	Loss-of-function	Loss-of-function	Loss-of-function

The asterisk in p.Leu780* denotes a premature termination (stop) codon.

Overall, all four patients showed sustained clinical benefit throughout the extended follow-up period.

As the patient-specific SCN1A variants had not previously been functionally characterized, we next investigated their expression, localization, and electrophysiological properties in HEK293 cells.

Western blot analysis confirmed the expression of the three missense variants (D1288Y, Y202D, and C1588R), whereas the truncating variant L780* was not detectable, consistent with markedly reduced or absent protein expression expected for a premature stop variant (Figure 1A). mScarlet served as a transfection control, with *SCN1A* detected at 300 kDa and the SPATPase at 113 kDa. Immunofluorescence microscopy demonstrated substantial plasma membrane localization of the detectable missense variants. This was supported by co-staining with the sodium-potassium ATPase (SPATPase), a known plasma membrane marker, showing colocalization with the *SCN1A* channels. The mean Pearson correlation coefficient (R) for colocalization with SPATPase was 0.80 ± 0.01 for CTL, 0.82 ± 0.02 for patient 1, 0.8 ± 0.03 for patient 2, patient 3 was not detectable and 0.86 ± 0.02 for patient 4, demonstrating a high degree of colocalization with the plasma membrane marker (Figure 1B). Conversely, attempts to colocalize the *SCN1A* channels with the endoplasmic reticulum (ER) marker calnexin were unsuccessful, with mean Pearson R values of 0.48 ± 0.04 for CTL, 0.44 ± 0.04 for patient 1, 0.50 ± 0.01 for patient 2, and 0.44 ± 0.04 for patient 4 (Figure 1C). These lower Pearson R values suggest poor colocalization with calnexin, providing no evidence for prominent retention of the detectable missense variants within the endoplasmic reticulum.

**FIGURE 1:**
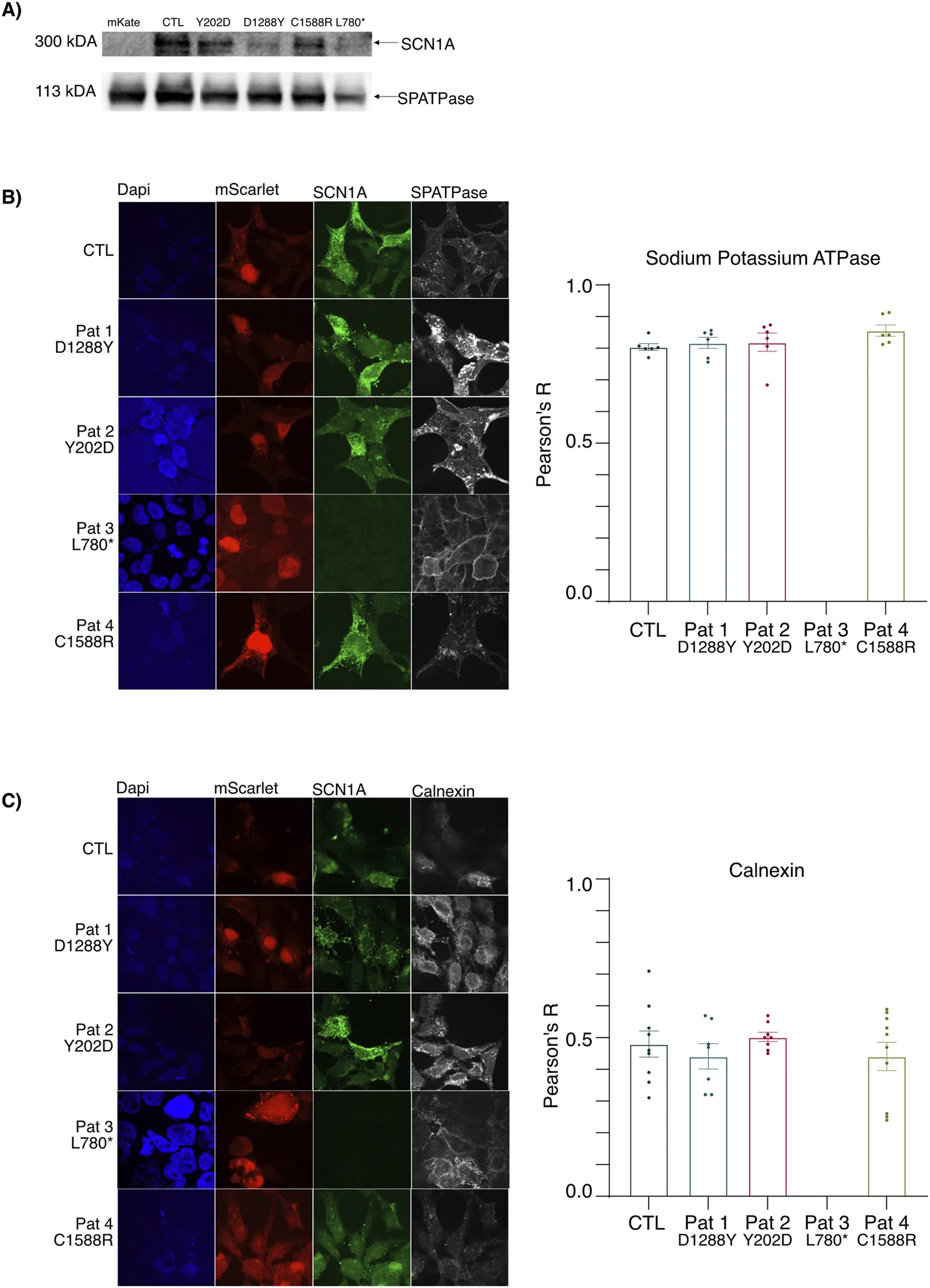
Expression and Subcellular Localization of SCN1A Variants. **(A)** Representative Western blot demonstrating expression of WT SCN1A and the three missense variants (Y202D, D1288Y, C1588R). The truncating variant L780* was not detectable. *SCN1A* is detected at ∼300 kDa, while SPATPase (∼113 kDa) serves as a loading control. **(B)** Immunofluorescence microscopy analysis showing localization of *SCN1A* (green) in control and patient cells with the indicated mutations. DAPI (blue) stains nuclei, mScarlet (red) is used as a transfection control, and SPATPase (gray) marks the plasma membrane. The graph on the right quantifies the colocalization of *SCN1A* with SPATPase using Pearson’s R values, showing high colocalization across conditions (CTL and mutant cells), demonstrating substantial colocalization with the plasma membrane marker. **(C)** Immunofluorescence microscopy with calnexin (gray), an endoplasmic reticulum (ER) marker, demonstrating localization of *SCN1A* in patient cells. The plot on the right shows lower Pearson’s R values for colocalization with calnexin, showing lower colocalization with the ER marker calnexin and providing no evidence for prominent ER retention of the detectable missense variants. Image acquisition and colocalization analysis were performed blinded using samples from three independent cell cultures. Each data point represents one coverslip averaged from 2 to 3 fields of view.

Electrophysiological properties of the *SCN1A* variants were assessed using whole-cell patch-clamp recordings from *SCN1A* WT or mutant transfected HEK293 cells. Sodium currents were induced by applying membrane depolarization steps from −90 mV to +60 mV for 500 ms. The four SCN1A variants exhibited loss-of-function phenotypes of varying severity. The L780, D1288Y, and C1588R variants displayed current densities of 17% or less compared with WT SCN1A, whereas Y202D retained approximately 62% of WT current density (Figures 2A,B).

**FIGURE 2:**
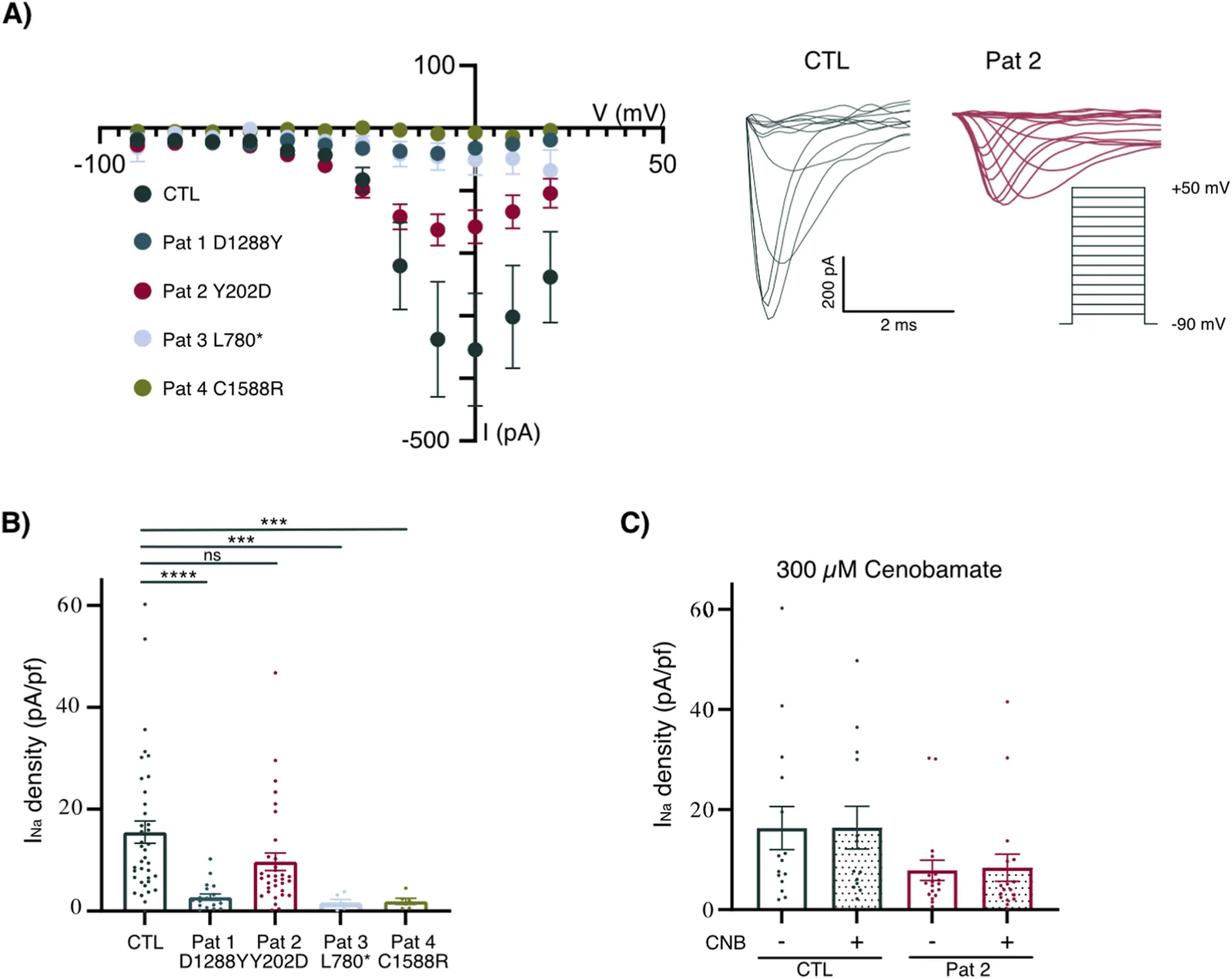
Expression and electrophysiological analysis of SCN1A variants. **(A)** Sodium current traces from whole-cell patch-clamp recordings of HEK293 cells transfected with either wild-type SCN1A (CTL; n = 38 cells) or one of four patient-derived SCN1A variants (Patient 1: p.Asp1288Tyr, n = 20; Patient 2: p.Tyr202Asp, n = 33; Patient 3: p.Leu780*, n = 3; Patient 4: p.Cys1588Arg, n = 7). Sodium currents were elicited by membrane depolarization steps from −90 mV to +60 mV for 500 ms. Representative current traces for the control (CTL) and Patient 2 are shown on the right. The graph on the left shows current–voltage (I–V) relationships. **(B)** Quantification of sodium current densities at 0 mV. Patient 1 (p.Asp1288Tyr), Patient 3 (p.Leu780*), and Patient 4 (p.Cys1588Arg) exhibited current densities of 17% or less compared to WT *SCN1A*. Patient 2 (p.Tyr202Asp) showed a partial loss of function with ∼62% of WT current density. Data are presented as mean ± SEM. Each data point represents one recorded cell. Statistical significance was assessed using the Kruskal–Wallis test followed by Dunn’s multiple-comparisons test. Adjusted P values *versus* WT were: Patient 1, P < 0.0001; Patient 2, P = 0.1954; Patient 3, P = 0.0004; and Patient 4, P = 0.0006. **(C)** Effect of acute 300 μM CNB exposure on peak sodium current density in WT and Patient 2 (p.Tyr202Asp) channels. Measurements were obtained from the same cells before and after CNB application. Paired recordings were available for 13 WT cells and 17 Y202D cells. Acute CNB exposure did not significantly alter peak sodium current density in either WT or Y202D channels under the experimental conditions used.

Our electrophysiological experiments were designed to determine whether the four patient-derived SCN1A variants represent loss-of-function (LoF) mutations. As peak sodium current amplitude is the primary and most robust parameter to identify LoF, we focused on peak sodium currents. Additional gating parameters were beyond the scope of this study.

We also investigated whether acute CNB exposure altered peak sodium current density in WT and Y202D SCN1A channels. First, we found that 300 μM CNB treatment of *SCN1A* WT expressing HEK293 cells did not alter response amplitudes to depolarization protocols when compared to control bath solution. Second, we tested the effect of acute CNB treatment on the partial loss of function mutant *SCN1A* Y202D, again yielding to no significant effects on peak currents (Figure 2C). These results indicate that acute CNB exposure did not measurably alter peak sodium current density in WT or Y202D channels under the experimental conditions used.

## Discussion

We previously reported a successful treatment of 4 patients with DS with CNB, with a follow-up time of 18 months (Makridis et al., 2022b). Here we extended these observations to a median follow-up of 38 months, showing sustained clinical benefit in all patients who remained on treatment. As our study included only cenobamate-responsive patients, future studies including non-responders will be required to determine whether SCN1A functional properties or other clinical factors predict treatment response.

The variants in the SCN1A gene identified in these patients were predicted by *in silico* analysis to be LoF variants. Our electrophysiological data confirm this prediction. The electrophysiological findings confirmed a loss-of-function phenotype for all four patient-specific SCN1A variants based on markedly reduced peak sodium currents. Although additional alterations in channel kinetics may contribute to the functional consequences of SCN1A LoF variants, these parameters were not assessed in the present study. The detectable missense variants showed preserved membrane localization, suggesting that their reduced sodium currents are primarily attributable to altered channel function rather than grossly impaired trafficking. In contrast, the truncating L780* variant was not detectable, consistent with markedly reduced protein expression.

The primary aim of our electrophysiological analysis was to determine whether the patient-specific SCN1A variants represent loss-of-function variants. Peak sodium current amplitudes provide a robust measure for this classification and confirmed a loss-of-function phenotype for all four variants. Additional electrophysiological properties, including channel kinetics, persistent sodium currents, and voltage- or use-dependent effects, were not assessed and remain subjects for future investigation. Furthermore, the CsF-based internal solution suppresses persistent sodium currents, and the physiological relevance of the 300 µM CNB concentration remains uncertain. Consequently, our acute CNB experiments should be interpreted as assessing effects on peak sodium current density under the experimental conditions used.

Although sodium channel blockers are generally considered contraindicated in DS, accumulating clinical evidence suggests that this may not apply uniformly to all sodium channel-targeting ASMs. Beneficial effects have been reported for selected agents, including lamotrigine and phenytoin, in individual patients or specific clinical situations (Dalic et al., 2015; Zographos et al., 2022; Abi Tayeh et al., 2024; Guerrini et al., 2021; Guerrini, 2012; de Lange et al., 2018). Our findings add to the growing clinical experience suggesting that selected sodium channel-targeting ASMs may provide clinical benefit in carefully selected patients with DS. However, larger prospective studies will be required before broader conclusions regarding the use of CNB in DS can be drawn.

Our clinical observations, supported by functional characterization of the underlying SCN1A variants, suggest that CNB may represent a therapeutic option for selected adults with genetically confirmed DS. However, given the small sample size and the inclusion of only treatment responders, these findings should be interpreted with caution until confirmed in larger prospective studies.

## Conclusion

This study showed that all four patient-specific SCN1A variants associated with DS exhibited a loss-of-function phenotype of varying severity. Acute CNB exposure did not alter peak sodium currents under the experimental conditions used. These findings further expand the clinical experience with CNB in adults with genetically confirmed DS. Prospective controlled studies are warranted to better define the efficacy and safety of CNB in DS.

## Data Availability

The raw data supporting the conclusions of this article will be made available by the authors, without undue reservation.
