# Supplementary material for: Cenobamate in Dravet syndrome with electrophysiologically confirmed SCN1A loss-of-function variants: long-term real-world follow-up
Source: Front Pharmacol. 2026 Sep 23;17:1894717. doi: 10.3389/fphar.2026.1894717 (PMC13643072; doi:10.3389/fphar.2026.1894717)
Supplement: Supplementary file 1 [file DataSheet1.pdf]

Supplementary Figure S1

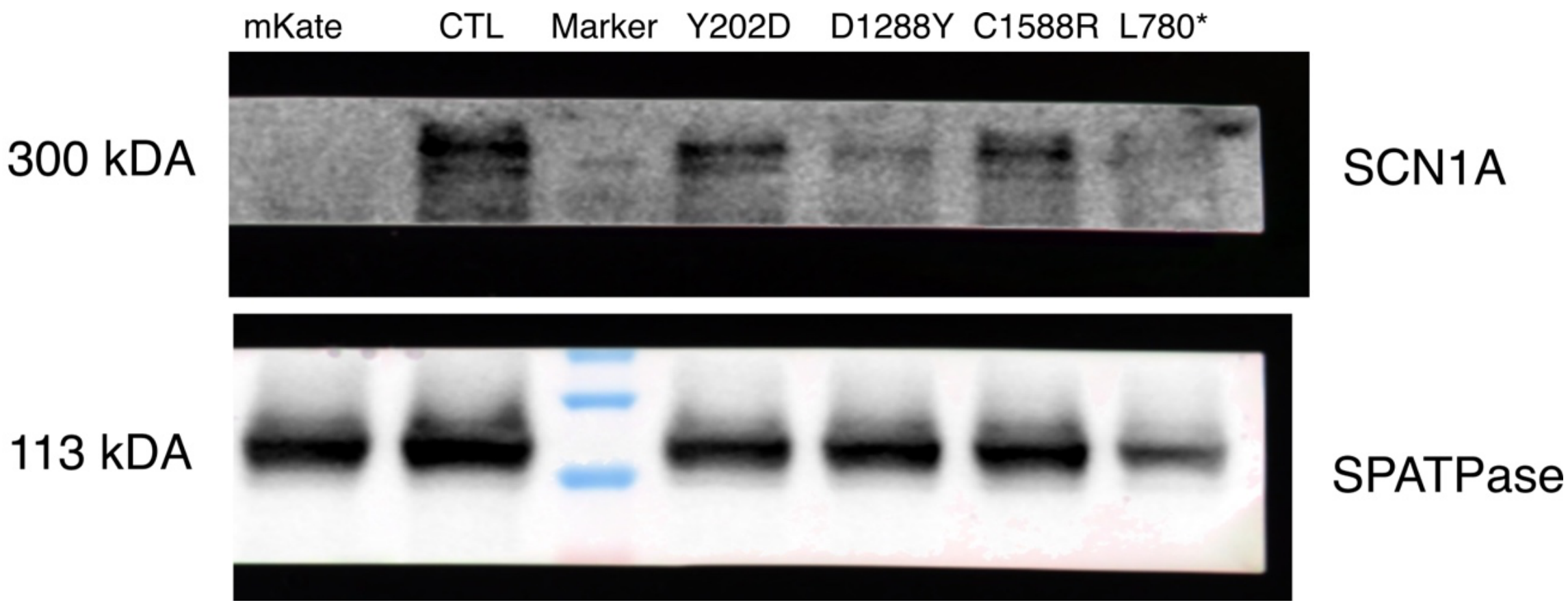

Uncropped Western blot images corresponding to Figure 1A. Uncropped images of the membrane strips used for detection of SCN1A (~300 kDa) and sodium-potassium ATPase (SPATPase; ~113 kDa) in HEK293T cells expressing WT SCN1A (CTL) or the indicated patient-derived SCN1A variants. The mKate empty-vector condition served as a negative transfection control. The membrane was physically cut into separate molecular-weight regions prior to antibody incubation. The images shown represent the complete, uncropped membrane strips used for antibody detection.
